# Sustained Benefit of Short-Term Levodopa Treatment on Inner Retinal Function in Patients With Diabetes

**DOI:** 10.1167/tvst.14.9.5

**Published:** 2025-09-02

**Authors:** Francesca Moore, Stephen Phillips, Dane Rubenstein, Caleigh Cullinan, Christina Young, Andrew J. Feola, Xiaxian Ou, Xiangqin Cui, Risha Patel, Mary Rhee, Andrew Hendrick, Machelle T. Pardue

**Affiliations:** 1 VA Center for Visual and Neurocognitive Rehabilitation, Atlanta VA Healthcare System, Decatur, Georgia, USA; 2 Department of Biomedical Engineering, Georgia Institute of Technology and Emory University, Atlanta, Georgia, USA; 3 Department of Ophthalmology, Emory University, Atlanta, Georgia, USA; 4 Department of Biostatistics and Bioinformatics, Rollins School of Public Health, Emory University, Atlanta, Georgia, USA; 5 Department of Medicine, Division of Endocrinology, Emory University, Atlanta, Georgia, USA

**Keywords:** diabetic retinopathy (DR), dopamine, diabetes, electroretinograms (ERGs), oscillatory potentials (OPs), levodopa (L-DOPA)

## Abstract

**Purpose:**

This study investigated the long-term progression of oscillatory potential (OP) implicit times (ITs) in individuals with preclinical diabetic retinopathy (DR) with and without levodopa (L-DOPA) treatment by quantifying functional and structural retinal changes.

**Methods:**

Participants from the Motz et al. (2020) study were re-evaluated after 5 years, including individuals with diabetes mellitus (DM) who received L-DOPA treatment for 2 weeks (the DM + L-DOPA group; *n* = 14), those who did not (the DM group; *n* = 6), and non-diabetic healthy controls (the control group; *n* = 37). Retinal function and structure were assessed using dim-flash electroretinography (ERG) and optical coherence tomography (OCT).

**Results:**

After 5 years, OP 1 and OP 2 ITs showed no significant differences among the groups (*P* > 0.05). The DM + L-DOPA OP IT values remained improved compared to baseline. The outer region thickness of the outer plexus layer (OPL) and ganglion cell layer (GCL) were significantly thinner in the DM + L-DOPA group compared to the DM group (*P* < 0.05). The DM group showed strong correlations between OP IT and OCT thickness across all retinal regions, whereas the DM + L-DOPA group correlations were similar to the control group.

**Conclusions:**

Short-term L-DOPA treatment led to significant functional improvements after 2 weeks, with trends suggesting sustained benefit over 5 years. Inner retinal structural differences suggest potential long-term benefit of L-DOPA on retinal health. These findings support OP IT delays as early biomarkers for preclinical DR and suggest L-DOPA may provide lasting neuroprotective benefits.

**Translational Relevance:**

Retinal dysfunction and inner retinal structural changes could be potential biomarkers for preclinical DR, and L-DOPA treatment may provide sustained benefits for the diabetic retina.

## Introduction

Diabetic retinopathy (DR) is currently the leading cause of blindness for working-age adults in the United States.^1^ In the United States, 9.6 million people are living with DR, and 1.84 million have vision-threatening DR.^2^ Globally, in 2020, it was reported that 103.12 million people have DR and that number is predicted to increase by more than 50% by 2045.^3^

DR is clinically diagnosed by identifying vascular lesions in the retina from clinical examination. Vascular lesions, such as microaneurysms, hemorrhages, and hard exudates, typically appear long after neuronal deficits in the diabetic retina have developed.^4^ Preclinical evidence for early neuronal changes in the diabetic retina includes alterations in retinal function, glial cell reactivity, glutamate regulation, microglial activation, and increased neuronal apoptosis.^5^^–^^9^ There is typically no noticeable vision loss at the early stages of retinopathy. However, as the disease progresses, patients are at risk of severe visual impairment or blindness. For this reason, the American Diabetes Association recommends that patients with diabetes receive eye examinations annually.^10^ However, according to the Centers for Disease Control and Prevention (CDC), 60% of adults diagnosed with diabetes do not get annual eye examinations.^11^

The current treatments for DR, such as anti-vascular endothelial growth factor (anti-VEGF), are administered at later stages of the disease after visual impairment has often occurred. Although anti-VEGF treatment has demonstrated efficacy in managing macular edema and proliferative DR, it is costly, invasive, and not consistently effective in improving vision loss. Some patients experience no significant improvements in visual acuity and may have an increased risk of ocular complications.^12^^–^^15^ Alternative treatment options include laser photocoagulation and vitreoretinal surgery; however, these procedures are invasive and can be detrimental to vision and retinal function.^16^^–^^18^ Therefore, there is a strong incentive to develop early detection methods and treatments targeting neuronal alterations in the retina, which precede vascular changes.

The electroretinogram (ERG) is a well-established clinical test used to assess retinal function that has become an increasingly valuable tool for understanding the underlying mechanisms of DR. In addition, ERG diagnostic devices, such as the RETeval, have made tracking ERG changes in diabetic retinas more convenient and efficient.^19^^,^^20^ ERG recordings are performed in the clinic following the International Society for Clinical Electrophysiology of Vision (ISCEV) standard which recommends dim- (−2 log cd · s/m2) and bright-flash (0.5 log cd · s/m2) stimuli with only the bright flash used for oscillatory potential (OP) analysis.^21^ In patients with DR, OP implicit time (IT) in response to this bright-flash stimulus is delayed compared to non-diabetic controls.^22^ Our previous study has shown that the ISCEV standard dim-flash is not strong enough to elicit measurable OPs in individuals with diabetes.^23^ However, our laboratory has found that a novel early marker for DR is a delay in OP IT in response to dark-adapted dim-flash (1.13 troland seconds) stimuli that specifically targets rod photoreceptors.^23^^,^^24^ We have shown that these OP IT delays occur very early after hyperglycemic induction in animal models of type 1 and type 2 diabetes.^23^^–^^27^ Human and animal studies from our laboratory and others have also demonstrated that a delay in OP IT precedes vascular changes in the retina with diabetes.^22^^,^^23^^,^^28^^,^^29^ A few studies have indicated that the OP or ERG IT delays progressively worsen as the retinopathy advances.^22^^,^^30^ However, little is known about how OP IT delays progress in individuals with diabetes, as well as how OP IT relates to late-stage vascular lesions.^20^^,^^23^

Identifying an early biomarker for preclinical DR, provides an early treatment window for interventions. Dopamine (DA) supplementation has been previously investigated as a potential neuroprotective agent to prevent the progression of DR due to a reduction of DA in the diabetic retina.^26^^,^^31^ Levodopa, a DA precursor, has been shown to improve OP IT delays in diabetic animals and in humans.^20^^,^^24^^,^^26^^,^^27^^,^^31^ We previously reported that 2 weeks of levodopa (L-DOPA) treatment had restorative effects on delayed OP IT in people with type 2 diabetes.^20^ However, it remains unclear whether ameliorating these early neuronal deficits can prevent late-stage vascular lesions and impact vision in DR. Tracking functional ERG changes alongside structural analysis has proven more effective in guiding treatment decisions and assessing disease progression than ERG alone.^32^

To determine whether early retinal neuronal changes preceded structural changes, retinal thickness abnormalities have been investigated using spectral domain optical coherence tomography (OCT). Various studies have investigated changes in retinal thickness between non-diabetic eyes and different stages of DR. Results have shown that inner retinal thickness decreases in early DR eyes and increases in advanced DR eyes that are absent of diabetic macular edema (DME), whereas outer retinal thickness is not as affected.^33^^–^^37^

This study examined (1) the longitudinal progression of OP ITs in individuals with DR, (2) the duration of short-term L-DOPA administration effects on eyes with DR, and (3) whether early neuronal dysfunction is associated with retinal structure as measured by OCT. To achieve this, we re-evaluated participants from the Motz et al. (2020) study, including individuals with diabetes who had received L-DOPA treatment for 2 weeks, those who had not, and a non-diabetic control group, using both ERG and OCT assessments.

## Materials and Methods

### Participants and Experimental Design

This clinical trial was registered with ClinicalTrials.gov (NCT05132660) and conducted in compliance with the Emory University Institutional Review Board, including obtaining informed consent. This study followed up with veteran participants with diabetes after 4.5 to 6 years who initially had no clinical signs of DR, but exhibited OP IT delays at baseline.^20^ Participants were previously screened via ERG assessment, and those with OP ITs exceeding the 95% confidence intervals (CIs) of a non-diabetic control group were randomly assigned to either a high-dose (50 mg carbidopa/200 mg L-DOPA) or low-dose (25 mg carbidopa/100 mg L-DOPA) of Sinemet.

Participants were originally recruited from the Atlanta Veterans Affairs (VA) Eye Clinic based on their diabetic teleretinal screening fundus photographs taken within the previous 6 months. Eligibility criteria required the absence of cataracts exceeding 1+ nuclear sclerosis or any signs of retinopathy. Additionally, participants were excluded if they had a DA-dysregulating disease or received DA agonists. ERG recordings and fundus images were obtained at baseline, following 2 weeks of L-DOPA treatment, and after a 2-week washout period. A small group of participants with diabetes and no clinical signs of DR, who underwent baseline measurements but did not receive L-DOPA treatments, were re-evaluated at the 4.5 to 6 year time point. Of the original 44 participants with diabetes, a substantial number of them were unable to return for re-evaluation due to factors such as relocation, mortality, cognitive impairment, lack of interest, or the development of visually significant cataracts (*n* = 24). Within the original diabetic cohort, the groups were labeled as DM participants with delayed OPs and DM participants with normal OPs. The mean and SD of Hba1c % levels of the DM participants were similar (delayed OPs = 7.30 ± 1.06 and DM normal OPs = 7.36 ± 1.01). Additionally, there was no DR present in any subject during the original study. The current cohorts do accurately represent the original cohorts. A non-diabetic control group with no history of ocular disease was included for comparison. Informed consent was obtained from all subjects after an explanation of the study. Experimental groups consisted of returning patients with diabetes mellitus (the DM group) without treatment (*n* = 6), patients with DM who were treated with L-DOPA for 2 weeks (the DM + L-DOPA group; *n* = 14), and non-diabetic control participants (the control group; *n* = 37). The low and high dose Sinemet treatment groups of the previous study were pooled into the DM + L-DOPA group due to a small sample size. Prior to analysis, data were screened for outliers using the ROUT method with a Q value of 5%.^38^ One subject was excluded due to OP amplitudes being an extreme statistical outlier.

### ERG Testing

ERGs were recorded using a handheld ERG device (RETeval; LKC Technologies, Inc., Gaithersburg, MD, USA). Skin preparation and the protocol for ERGs were described previously.^20^ Briefly, the skin under each eye was cleaned with a cotton applicator (Nuprep Skin Prep Gel; Weaver and Company, Aurora, CO, USA), followed by an alcohol wipe. Once the skin dried, sticker electrodes (RETeval Sensor Strips; LKC Technologies, Inc., Gaithersburg, MD, USA) were applied as recommended. The participants were dark adapted for 10 minutes and dim red LED illumination was used to position the participant and Ganzfeld.

ERG recordings consisted of presenting a dim-flash (1.13 troland seconds, interstimulus time of 5 seconds) and a bright flash (85 troland seconds; interstimulus time of 30 seconds) to one eye. The protocol was then repeated on the contralateral eye. For the 5-year visit, additional recordings were made for the dim-flash to enhance the OP signals (*n* = 18 intra-step repeats and 3–4 inter-step repeats).

### ERG Analysis

Our analysis builds upon our previously established approach. All waveforms from the three visits were reanalyzed under the same conditions and filtering parameters. Waveforms were processed with custom software (ERGAssist).^39^ Briefly, repeated ERG signals were averaged, and the resulting waveforms were smoothed using one-dimensional denoising via a wavelet filter. The waveform was passed through a low-pass filter (85 hertz [Hz]) to identify the location of the b-wave, with the peak amplitude labeled as the b-wave and the corresponding time as the b-wave IT. The a-wave was identified through a multi-step process due to signal variability. Initially, the waveform was processed with a Hampel filter to remove outliers and further smoothed using a Savitzky-Golay filter. Local minimum values between the initiation of the flash stimuli and the b-wave were identified on this smoothed waveform. These local minima were then mapped onto the original waveform to accurately locate the a-wave. If multiple local minima were detected, the earliest minimum that appears in time on the low-pass filtered waveform was designated as the a-wave amplitude and IT. Subsequently, the OP signal was isolated by passing the averaged ERG waveform through a fifth-order Butterworth bandpass filter between 85 and 190 Hz. The first OP is marked as the first positive peak after the a-wave trough, with the subsequent OPs (OPs 2–5) marked sequentially.

The quality of the OP response was objectively assessed using the ERGAssist program, which calculates the maximum amplitude, variance, and standard deviation of the OP waveform in the pre-stimulus (pre-flash) region.^39^ To qualify for analysis, the OP waveform between the a-wave and b-wave was required to exhibit an amplitude, variance, and standard deviation at least two, five, and two times greater, respectively, than the pre-stimulus region. Additionally, the average power of the OP waveform in this interval needed to exceed 0.35 microvolts, a threshold determined empirically. These criteria ensured that the OP response was distinguishable from the recorded baseline variation and noise preceding the flash.

Delayed OP ITs were determined by comparing to the 95% CIs from control subjects without diabetes (the control group). The control group consisted of one eye per control participant from Motz et al. 2020 (*n* = 10) and additional control participants (*n* = 27). These ERGs were reanalyzed as described above and 95% CIs were calculated.

Data were collected from both eyes of each participant. However, only one eye from each participant was used for analysis based on the quality of the ERG signals acquired under dim-flash stimulation. Although subsequent bright ERG and OCT analyses were evaluated independently, eye selection determined by the dim-flash recordings remained consistent. If the selected eye produced a high-quality dim-flash signal but did not meet quality thresholds for the bright flash, the bright flash data for that eye were excluded rather than switching to the contralateral eye. This ensures that all analyses across time points were consistently performed on the same eye, thus minimizing variability and enhancing the reliability of longitudinal comparisons.

Statistical analyses were conducted to compare IT and amplitudes across groups and time points. Due to the nonparametric nature of the data, the Wilcoxon rank-sum test applied for unpaired comparisons between the DM and DM + L-DOPA groups. To control for multiple comparisons, the Holm-Sidak correction was applied. For within-group comparisons across multiple time points, a 1-way repeated measures ANOVA was conducted. Given that the assumption of sphericity was violated, a Greenhouse-Geisser correction was applied to adjust the degrees of freedom. All analyses were performed using GraphPad (GraphPad Software, version 10.2.3(347); GraphPad, LLC, Boston, MA, USA).

### OCT Segmentation

Macular OCT scans (20 degrees × 20 degrees, 49 B-scans) were recorded using the Spectralis HRA + OCT (Heidelberg Engineering, Heidelberg, Germany). Retinal layers and Early Treatment Diabetic Retinopathy Study (ETDRS) grid regions of each layer were analyzed for differences in retinal thickness mean and standard deviation across groups at the year 5 time point. As OCTs were not completed in the previous study, OCT data were not available for earlier time points. Of the 20 subjects with diabetes with reliable ERG data, 4 of them were excluded from OCT analysis due to the presence of DME, vitreomacular traction (VMT), or low-quality scans. Of the 37 participants used for control data, only 27 of them had OCT scans performed. Two of the control participants were removed due to low-quality scans (off-centered, movement artifacts, and breakages). The 95% CIs from the control group were used for comparison. The eye that was chosen for the ERG data analysis remained consistent for the OCT analysis.

**Figure 1.**
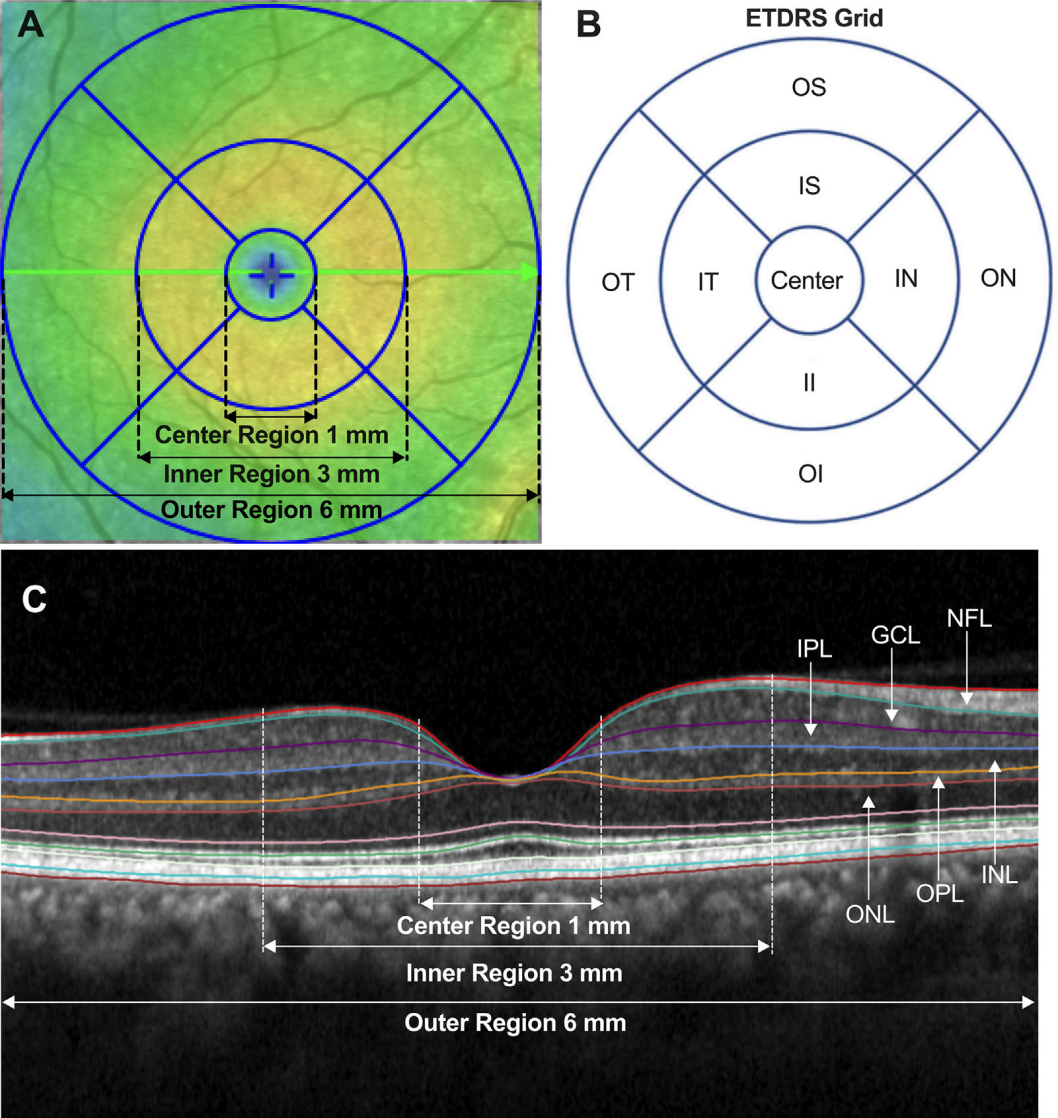
OCT segmented retinal layers. (**A**) En face macular thickness map showing the 1 mm center region, 3 mm inner region, and 6 mm outer region. (**B**) Standard ETDRS grid showing the 1 mm center, 3 mm inner region average of the four parafoveal subfields (inner superior [IS], inner nasal [IN], inner inferior [II], and inner temporal [IT]), and 6 mm outer region average of the four perifoveal subfields (outer superior [OS], outer nasal [ON], outer inferior [OI], and outer temporal [OT]). (**C**) Macular OCT B-scan with Heidelberg segmented retinal layers followed by manual correction. The segmented layers are the nerve fiber layer (NFL), ganglion cell layer (GCL), inner plexus layer (IPL), inner nuclear layer (INL), outer plexus layer (OPL), and outer nuclear layer (ONL).

All B-scans were automatically segmented using the Heidelberg Eye Explorer segmentation algorithm followed by a manual correction, as indicated in Figure 1. The retinal layers segmented were the nerve fiber layer (NFL), ganglion cell layer (GCL), inner plexiform layer (IPL), inner nuclear layer (INL), outer plexiform layer (OPL), and outer nuclear layer (ONL; see Fig. 1). The mean thickness of the full retina and each layer were computed using the three rings of the ETDRS grid: a central foveal 1-mm diameter ring, pericentral 3-mm inner ring, and paracentral 6-mm outer ring. The inner and outer rings were further divided into temporal, superior, nasal, and inferior regions. The differences between DM + L-DOPA group and the DM group for mean retinal thickness within each retinal layer and ETDRS region were evaluated using a nonparametric Mann-Whitney *U* test. *P* values reported were two-tailed.

To evaluate the correlation between retinal thickness and retinal function, Spearman's rank correlation tests were applied to the thickness of each OCT layer and ETDRS region compared to each ERG IT parameter.

### Fundus Photography

Fundus photography was conducted on a CR-2 PLUS AF Digital Retinal Camera (Canon, Tokyo, Japan). Three views per eye were obtained: the macula, optic nerve head, and superior nasal area. The lights in the examination room were dimmed to increase the pupil diameter but the participants’ eyes were not dilated. Fundus photographs were evaluated by an ophthalmologist for signs of vascular pathology.

### Effects of Treatment and Covariates on ERG and OCT Outcomes

A1C, body mass index (BMI), and age values were collected from patient charts over the course of the study period, encompassing all recorded measurements from the baseline visit through the 5-year follow-up visit. Because individual subjects did not have A1C and BMI measurements taken on the exact dates of their study visits, these values were averaged across all available time points within the study period, ensuring representation of their metabolic activity.

A series of mixed-effects models were used to evaluate the effects of treatment status (DM versus DM + L-DOPA), time point (baseline versus week 2 versus year 5), and their interactions on the estimated a-wave, b-wave, and OP IT (week 2 × DM + L-DOPA, year 5 × DM + L-DOPA, and year 5 × DM). We included demographic factors, such as age, average A1C, A1C duration, and average BMI, as covariates in our analysis. These models were subsequently applied to a separate analysis examining OCT measurements at year 5 to assess retinal layer thickness.

Across all models, none of the main effects (treatment, timepoint, or demographic) nor their interaction terms reached statistical significance (*P* values > 0.05). The inclusion of age, average A1C, A1C duration, or average BMI as additional predictors did not significantly alter the results. No evidence of differential effects across time points was observed, as none of the interaction terms yielded significant findings. These results indicate that treatment status, time point, and demographic factors, such as age, A1C, and BMI, did not demonstrate a measurable influence on the outcomes.

## Results

### Dim-Flash OP Timing Shows Sustained Benefit From L-DOPA

**Figure 2.**
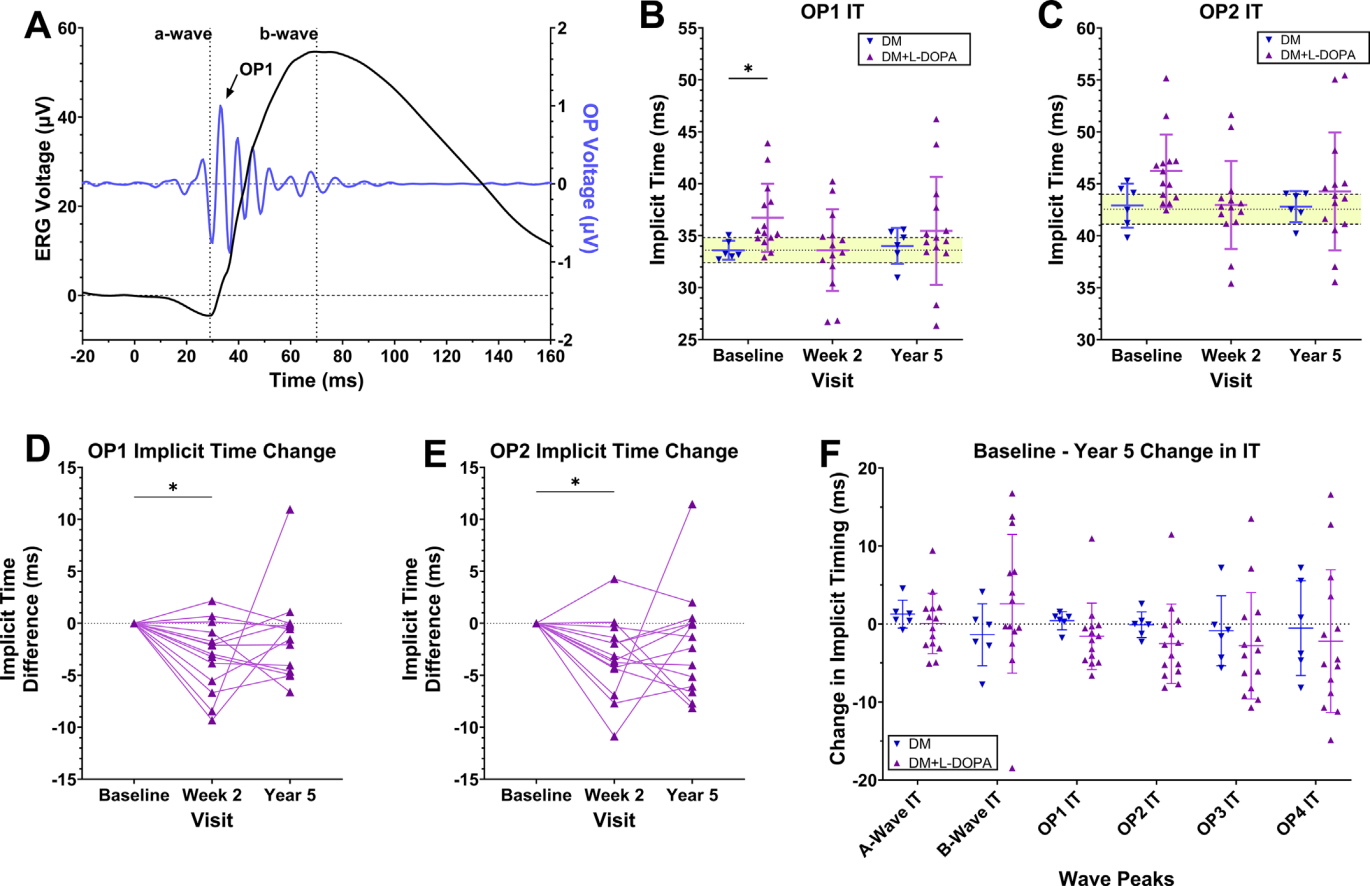
Averaged (±SD) ERG waveforms and OP ITs in DM + L-DOPA and DM groups at baseline, week 2, and year 5 exposed to dim-flash stimuli. (**A**) A representative ERG waveform (*black line*) with the filtered OP waveforms overlaid (*blue line*). The a-wave is indicated by the *vertical dotted line*. OP 1 is identified as the first peak following the a-wave trough. (**B, C**) IT for OP 1 and OP 2, respectively, across baseline, week 2, and year 5. The *yellow horizontal band* indicates the normative 95% confidence interval derived from healthy controls. A Wilcoxon rank-sum test comparing the baseline and year 5 visits between the DM and DM + L-DOPA groups was performed. OP 1 IT was significantly delayed in the DM + L-DOPA group compared to the DM group at baseline, as intended during the screening process. (**D, E**) Baseline corrected IT for OP 1 and OP 2 (1-way repeated measures ANOVA) comparing the DM + L-DOPA group across all three time points. Significant reductions in OP 1 and OP 2 IT were observed by week 2 in the DM + L-DOPA group, with improvements persisting through year 5, although not reaching statistical significance at that point. (**F**) The change in IT from baseline to year 5 for individual IT timings for the a-wave, b-wave, and OPs 1 to 4.

Figure 2A shows a representative ERG waveform in response to the dim-flash stimuli (1.13 troland seconds). In the Motz study, participants with DM were categorized into a delayed OP treatment group and a no-delay group based on their baseline OP response times. Given this classification, we expect that upon reanalysis the baseline DM + L-DOPA group should exhibit delayed OPs, whereas the DM group should not. As expected, the DM + L-DOPA group at baseline was identified as having significant OP 1 IT delays compared to the DM group under dim-flash conditions (1.13 troland seconds; Fig. 2B; DM: *n* = 6, OP 1 IT = 33.60 ± 0.91 ms; DM + L-DOPA: *n* = 14, OP 1 IT = 37.03 ± 3.23 ms; Wilcoxon rank-sum exact test, *P* = 0.017; see Fig. 2B). Although OP 2 IT did not reach statistical significance at baseline in this re-analysis, a similar trend was observed, with the DM + L-DOPA group exhibiting greater delays compared to the DM group (DM: *n* = 6, OP 2 IT = 42.90 ± 2.12 ms; DM + L-DOPA: *n* = 14, OP 2 IT = 46.80 ± 3.61 ms; Wilcoxon rank-sum exact test, *P* = 0.099; Fig. 2C). At week 2, OP 1 and OP 2 IT from the DM + L-DOPA group fell within the normative range derived from the control group of healthy participants (indicated by the highlighted yellow region). Importantly, at year 5, the average OP ITs were not significantly different between the groups for OP 1 or OP 2 IT (OP 1 IT: DM, *n* = 6, 34.01 ± 1.72 ms, DM + L-DOPA, *n* = 14, 35.46 + 5.19 ms, *P* = 0.602; OP 2 IT: DM = 42.80 + 1.50 ms; DM + L-DOPA = 44.27 ± 5.67 ms, *P* > 0.602; Wilcoxon rank-sum; see Figs. 2B, 2C).

Within the DM + L-DOPA group, an analysis of the differences in baseline compared with all time points (1-way repeated measures ANOVA) demonstrated significant reductions in OP 1 (*P* = 0.011) and OP 2 (*P* = 0.014) IT by week 2 (Figs. 2D, 2E). Importantly, at year 5, OP 1 and OP 2 ITs in the majority of individuals from the DM + L-DOPA group remained below baseline (Figs. 2D, 2E). These trends suggest that improvement in OP timings persisted in the DM + L-DOPA group for 5 years post-treatment. An analysis of dim-flash amplitudes across groups reveals no significant differences across baseline and year 5 for the a-wave, b-wave, and OPs 1 to 4 (Supplementary Fig. S1).

To assess ERG changes with the progression of diabetes, we compared the difference in ITs between baseline and year 5 results for all ERG waves (Fig. 2F). The DM group (*n* = 6) exhibited minimal shifts in ERG IT, with differences near zero from baseline to year 5 (e.g. DM OP 1 year 5 IT change = 0.41 ± 1.2 ms; OP 2 year 5 IT change = −0.10 ± 1.6 ms). Although no statistically significant differences were detected in OP IT, the DM + L-DOPA group exhibited a subtle trend toward faster OP IT measurements over the 5-year period; a trend we attribute to the L-DOPA treatment benefits seen at week 2 (see Figs. 2D, 2E). No significant differences in OP IT were detected between the DM + L-DOPA and DM groups between baseline and year 5 (OP 1 IT change, *P* = 0.180; OP 2 IT change, *P* = 0.275; OP 3 IT change, *P* = 0.776; and OP 4 IT change, *P* = 0.776; Wilcoxon rank-sum exact test).

When comparing ITs across groups under bright flash conditions (85 troland seconds), no significant differences were observed between the DM and DM + L-DOPA groups across all time points (DM: *n* = 6, DM + L-DOPA: *n* = 11; Wilcoxon rank-sum exact test, *P* > 0.05; Supplementary Fig. S2). Within the DM + L-DOPA group at different visits, the year 5 a-wave IT was significantly increased compared to week 2 (one-way repeated measure [RM] ANOVA: a-wave: *P* = 0.0017). Interestingly, the a-wave and OP ITs showed trends for slower ITs at year 5 (see Supplementary Fig. S2). An analysis of bright flash amplitudes across groups and over time revealed no significant differences (Wilcoxon rank-sum exact test, *P* > 0.05; Supplementary Fig. S3).

### Retinal Thinning in Diabetic Eyes That Received Sinemet Treatment

**Figure 3.**
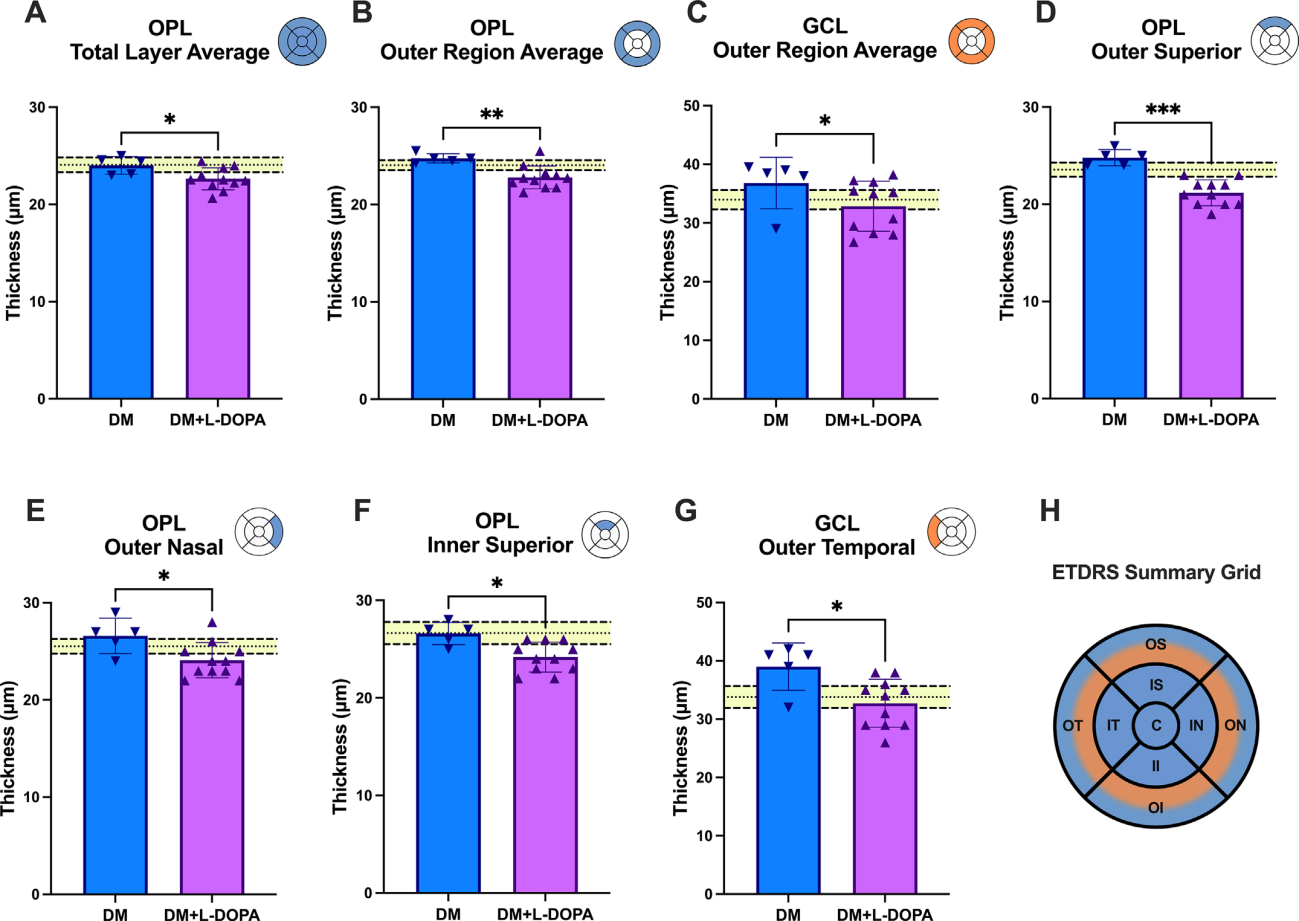
OCT retinal thickness at year 5 in participants with diabetes (DM, *n* = 5) and participants with diabetes who received L-DOPA treatment (DM + L-DOPA, *n* = 11). The *yellow highlighted* regions represent the normative 95% CI range from the healthy controls. (**A**) OPL total layer average. (**B**) OPL outer region average. (**C**) GCL outer region average. (**D**) OPL outer superior. (**E**) OPL outer nasal. (**F**) OPL inner superior. (**G**) GCL outer temporal (Mann-Whitney *U* test: *P* < 0.05 *, *P* < 0.01 **, *P* < 0.001 ***). (**H**) ETDRS Summary Grid of retinal thickness changes. *Blue* represents regions with OPL retinal thickness changes and *orange* represents regions with GCL retinal thickness changes.

Retinal layer thickness in DM + L-DOPA eyes were consistently thinner than DM eyes. The only retinal layer to show a significant difference between total layer averages was the OPL. As shown in Figure 3A, the DM + L-DOPA eyes (22.64 ± 1.13 µm) had a significantly thinner total OPL layer average thickness than DM eyes (24.02 ± 0.91 µm; *P* = 0.018). Within the OPL layer, the outer region was also significantly thinner in DM + L-DOPA (22.80 ± 1.18 µm) compared with DM eyes (24.75 ± 0.47 µm; *P* = 0.006; Fig. 3B). The outer region average of the GCL was also significantly thinner in the DM + L-DOPA group (32.86 ± 4.25 µm) compared to the DM group (36.80 ± 4.40 µm; *P* = 0.038; Fig. 3C).

The retinal quadrants in the OPL and GCL layers were significantly different. The OPL was thinner in DM + L-DOPA versus DM eyes in the outer superior, outer nasal, and inner superior regions (outer superior: DM + L-DOPA group = 21.18 ± 1.33 µm; DM group = 24.80 ± 0.84 µm; *P* < 0.001; outer nasal: DM + L-DOPA group = 24.09 ± 1.81 µm; DM group = 26.60 ± 1.82 µm; *P* = 0.031; inner superior: DM + L-DOPA group = 24.18 ± 1.54 µm; DM group = 26.60 ± 1.14 µm; *P* = 0.011; Figs. 3D–F). The outer temporal region of the GCL was also significantly thinner in the DM + L-DOPA group (32.73 ± 4.10 µm) compared to the DM group (39.00 ± 4.06 µm; *P* = 0.010; Fig. 3G). Data for each treatment group and each layer examined can be found in Supplementary Figures S4 and S5 and Supplementary Table S1.

### Diabetic State and L-DOPA Treatment Influence Associations Between Retinal Function and Structure

**Figure 4.**
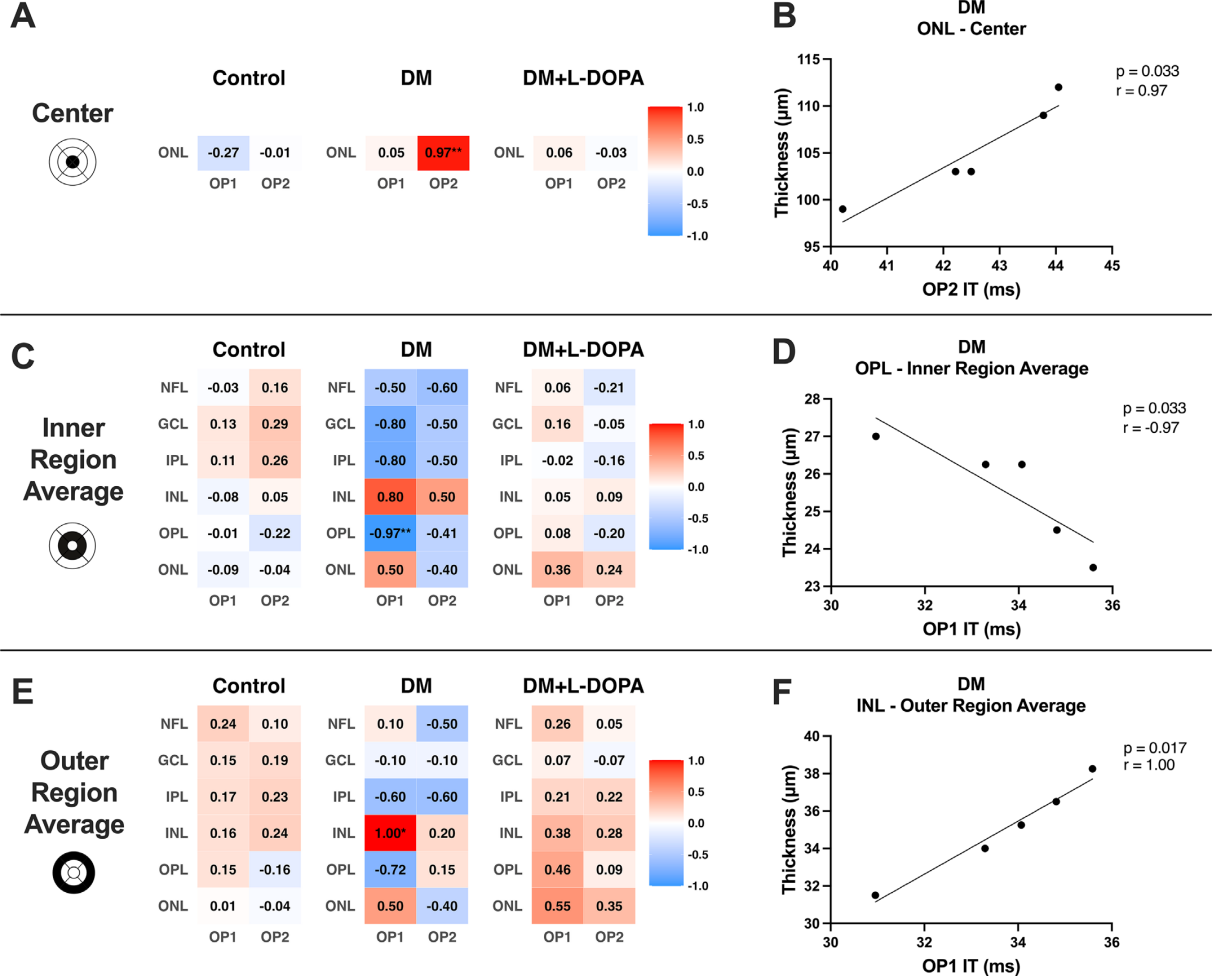
Spearman correlation heatmap displaying the correlation coefficient, r, between OCT regional retinal thickness and ERG OP 1 and OP 2 IT as defined by EDTRS regions. **(A**) Center, (**C**) inner, and (**E**) outer. Significant correlations for the DM group are plotted to the right of the heatmap for each region: (**B**) ONL versus OP 2 IT, (**D**) OPL versus OP 1 IT, and (**F**) INL versus OP 1 IT (*P* < 0.05 *, *P* < 0.01 **).

Correlations for dim-flash OP IT and OCT retinal layer thickness were investigated by plotting data of all groups together and separately. There were no statistically significant findings across all of the groups. However, our correlation analysis of dim-flash OP IT and OCT retinal thickness showed interesting differences between the center region versus the inner and outer regions, as well as differences in the general trends between the DM and DM + L-DOPA groups (Fig. 4).

Near the central region of the macula, only the ONL was evaluated (see Fig. 4A). The control and DM + L-DOPA were similar with weak correlations between retinal thickness and OP 1 and OP 2 ITs (the control group = OP 1 IT, *r* = −0.27; the DM + L-DOPA group = OP 1 IT, *r* = 0.06; the control group =, OP 2 IT, *r* = -0.01; and the DM + L-DOPA group = OP 2 IT, *r* = –0.03). In comparison, the DM group had a significant positive correlation with OP 2 IT (*P* = 0.033, *r* = 0.97; see Fig. 4B) and a weak positive correlation with OP 1 IT (*r* = 0.05).

Outside the macular region, we measured correlations in the inner region between retinal thickness and OP ITs (see Fig. 4C) were more similar in the control and the DM + L-DOPA groups, compared to the DM group. The control and DM + L-DOPA groups had weak correlations with both positive and negative trends (control group = OP 1 IT, *r* = 0.005 ± 0.10; DM + L-DOPA group = OP 1 IT, *r* = 0.12 ± 0.13; control group = OP 2 IT, *r* = 0.08 ± 0.19; and the DM + L-DOPA group = OP 2 IT, *r* = –0.05 ± 0.18). However, in the DM group, the correlations had overall negative trends with a significant negative correlation for OPL and OP 1 IT (*P* = 0.033, *r* = –0.97; see Fig. 4D). Additionally, moderate positive correlations were found for the INL and ONL with OP 1 and INL for OP 2.

The correlations in the outer region were similar to the inner region, with similarities between the control and the DM + L-DOPA groups versus the DM group. Weak to moderate positive relationships were found between the control and DM + L-DOPA groups between retinal thickness and ERG timings (control group = OP 1 IT, *r* = 0.15 ± 0.07; DM + L-DOPA group = OP 1 IT, *r* = 0.32 ± 0.17; the control group = OP 2 IT, *r* = 0.09 ± 0.16; and the DM + L-DOPA group = OP 2 IT, *r* = 0.15 ± 0.16; see Fig. 4E). In the INL of the DM group, the outer region average had a significant positive correlation with OP 1 IT (*P* = 0.017, *r* = 1.00; see Fig. 4F), whereas moderate negative trends were found for NFL, IPL, and OPL.

### DR Development Over 5 Years

All participants received fundus imaging at baseline and at the 5 year visit to evaluate for potential signs of retinal vascular changes with DM. Of the 6 participants in the DM group, 3 developed mild nonproliferative diabetic retinopathy (NPDR) by year 5 (50%). In contrast, of the 14 participants in the DM + L-DOPA group, 3 were identified to have mild NPDR, 1 with moderate NPDR, and 2 with DME at the 5 year visit (total of 43%; see the Table). We carefully reviewed the charts for possible confounding characteristics but did not find any notable characteristics with these participants that could account for this progression in clinically recognizable stages of DR.

**Table.** Characteristics of Returning Participants With Diabetes and No Treatment (DM) and With Diabetes and L-DOPA Treatment (DM + L-DOPA) from the Motz et al. 2020 Study at the 5 Year Visit and a Cohort of Non-Diabetic Control Participants

	Control	Diabetics Without Treatment (DM)	Diabetics With Treatment (DM + L-DOPA)
Patients, *n*	37	6	14
Sex, *n* of males	29	6	14
Age, y, mean ± SD	54.l ± 12.7	57.3 ± 12.9	69.9 ± 5.6
Disease duration, y, mean ± SD	—	18.2 ± 6.0	17.2 ± 8.l
Type of diabetes, *n* of type 2	—	4	14
HbAlc, %, mmol/mol, (mean ± SD)	5.65 ± 0.38 (38.3 ± 4.1)	8.36 ± 1.02 (67.9 ± 11.1)	7.75 ± 1.39 (61.2 ± 15.2)
Race	28 AA, 8 W	5 AA, 1 W	10 AA, 3 W
Ethnicity, *n* of Hispanic or Latino	1	0	0
BMI, mean ± SD	29.2 ± 5.7	28.8 ± 5.9	30.2 ± 5.5
Diabetic retinopathy			
Mild nonproliferative	—	3	3
Moderate nonproliferative	—	—	1
Diabetic macular edema	—	—	2

AA, African American; W, White.

## Discussion

In this study, we conducted a 5-year follow-up examination of patients originally recruited in Motz et al., 2020 and showed that rod-driven inner retinal function, as measured with dark-adapted, dim-flash OP IT, did not worsen over the 5-year period.^20^ In the DM + L-DOPA group, dim-flash OP IT remained similar to values measured after the 2 weeks of L-DOPA treatment. In the untreated DM group, dim-flash OP IT was comparable to the non-diabetic control group across all time points. Furthermore, we observed decreased OPL thickness in the DM + L-DOPA group compared to the untreated DM group, particularly in the inner and outer retinal regions, which correlated with OP IT values. The central region of the retina exhibited minimal structural changes. Furthermore, we found that correlations between retinal layer thickness and OP IT were most similar between the control group and the DM + L-DOPA versus the DM groups. Together, these findings reinforce the potential of dim-flash OP IT as an early functional biomarker for preclinical DR and suggest that L-DOPA may have lasting neuroprotective effects on the diabetic retina.

### Dark-Adapted Dim-Flash OP Delays Detect Early Signs of DR

A substantial amount of evidence shows retinal functional changes occur prior to vascular abnormalities in DR.^9^^,^^40^^,^^41^ However, DR assessments in the clinic do not currently utilize functional measurements, such as ERGs. The ERG deficits have been reported before a clinical diagnosis of DR and greater deficits develop with DR progression.^20^^,^^42^^–^^44^

The majority of ERG studies on DR progression have focused on cone-driven flicker stimuli amplitude and IT with decreased amplitudes and delayed timing of the flicker response.^42^^,^^45^^–^^48^ Other studies using dark-adapted full-field ERGs often focus on significant changes in a-wave and b-wave IT using the ISCEV standard flash stimuli. For instance, dark-adapted ERGs with an intermediate flash stimuli (0.81 cd s/m^2^) elicited an a-wave IT delay in patients with diabetes as compared with non-diabetic controls.^42^ When OP amplitudes are analyzed in studies with patients with diabetes, it is typically using the ISCEV recommendations of analyzing the OPs in response to bright flash stimuli.^49^^,^^50^ OP amplitudes have shown a significant, negative correlation with increasing severity of DR.^51^^,^^52^ Likewise, in other studies, decreased OP amplitudes and delayed ITs become progressively worse with each clinical stage of DR.^53^ Prior studies have also used the OP parameters in combination with fundus imaging to create a more robust detection of DR stages and develop a potentially more sensitive biomarker for progression.^19^^,^^32^ In the current study, we focused on OP IT delays in response to a dark-adapted dim-flash which targets predominately rod photoreceptor pathways. Our previous data in diabetic rodents and in clinical studies has shown that rod pathways are most susceptible to diabetes.^20^^,^^24^^,^^31^

Animal studies have shown that dim-flash OP IT delays can occur in type 1 and type 2 diabetes, prior to the appearance of vascular changes.^24^^,^^25^^,^^31^ In the type 1 streptozotocin (STZ) model of diabetes, dim-flash OP IT delays are detected within 1 to 2 months after diabetes induction in mice and rats before the appearance of acellular capillaries at 6 to 8 months.^25^^,^^31^ Additionally, the OP delays became progressively more delayed with the duration of diabetes.^23^ Similarly, in type 2 high fat diet models of diabetes, OP delays are detected after 6 months and precede vascular structural lesions.^43^

Based on the prior data, this study investigated the feasibility of using OP recordings as a biomarker for early-stage DR and whether OP ITs changed over a 5-year time period. The DM group was not randomized for treatment in the original Motz study because OP ITs were within the non-diabetic normative values (see Figs. 2B, 2C). The OP ITs in the DM group did not significantly change from baseline after 5 years, despite half of the group progressing to mild NPDR, with values still within the normative range. This may indicate that OP IT is slow to change in patients with diabetes. However, this would appear to contradict prior studies that have shown that OPs develop smaller amplitudes and slower ITs with each stage of DR.^22^^,^^30^ Thus, another possible explanation is that the individuals in the DM group were still not showing early signs of diabetic retinal dysfunction.

Although patients with diabetes, with and without clinically detectable DR, have demonstrated abnormalities in a-wave, b-wave, and OPs, the OPs are considered the most sensitive measure of dysfunction before retinal neovascularization.^23^^,^^25^^,^^54^ Our findings in patients with diabetes and rodents support this, as significant OP delays were observed in this study and past studies, whereas no significant a-wave and b-wave IT delays were detected under dim-flash conditions.^20^^,^^23^^–^^26^ In this study, analysis of bright flash IT and amplitude data showed that OPs remained within a normative healthy range, with no significant changes from baseline to year 5, despite prolonged diabetes duration.

### Acute L-DOPA Treatment had Prolonged Benefits to Inner Retinal Function in Diabetes

Previous work has shown L-DOPA to be beneficial in DR, likely due to deficits in retinal DA. Dopaminergic amacrine cells have been shown to degenerate 6 months after STZ induction in diabetic rodents.^31^^,^^55^^,^^56^ Much earlier changes in retinal DA turnover have been found in diabetic rodents after only 4 to 6 weeks of STZ-induced hyperglycemia, prior to any vascular changes.^24^^,^^31^ Furthermore, these studies found that using L-DOPA to treat the functional OP delays, either at the start of diabetic induction or after the OP delays first appeared in diabetic rodents, provided significant protection to the OP timing.^24^^,^^26^^,^^27^^,^^31^

We previously reported the benefits of acute L-DOPA treatment in patients with diabetes and no retinopathy.^20^ Patients with diabetes and dark-adapted OP delays in response to a dim-flash (1.13 troland seconds) were randomized to low-dose (25 mg carbidopa/100 mg L-DOPA or high-dose (50 mg carbidopa/200 mg L-DOPA) Sinemet. After only 2 days of low-dose L-DOPA treatment, OP 2 ITs were significantly faster and after 2 weeks of low and high-dose treatment, OP 2 ITs had improved to be statistically similar to non-diabetic control IT values. L-DOPA treatment was stopped, and ERGs were recorded again at 4 weeks post-treatment (after 2 weeks of washout). The benefits to OP 2 ITs were sustained during this 2-week washout period. Furthermore, we also evaluated OP ITs in response to bright flash stimuli (85 troland seconds) and found no significant differences between the Sinemet-treated patients with diabetes and non-diabetic control participants. These results suggest that L-DOPA treatment provides a sustained, acute, benefit to inner retinal function, beyond the treatment period.

In the current study, the same patients with DM from the Motz et al. study were re-evaluated 5 years after treatment. We found that most participants showed a reduction of OP ITs (see Figs. 2D, 2E). Remarkably, OP 1 IT remained below baseline for approximately 80% of the participants. These results suggest that even short-term L-DOPA treatment could have a sustained, long-term, effect on rod-driven inner retinal function. Alternatively, they could also indicate there is a slow progression of OP IT delays with DM.

Bright flash ERGs continued to show no significant differences in OP timings, with OP 1 and OP 2 remaining within the normative range. However, by year 5, the average OP IT exhibited a trend toward further delay. Notably, a-wave IT under bright flash conditions showed improvement at week 2 compared with baseline recordings, although this change did not reach statistical significance. By year 5, a-wave IT had significantly worsened relative to week 2 values, suggesting that although L-DOPA initially provided functional benefits, its long-term protective effects on a-wave timing under bright flash conditions may diminish over time.

### Benefits of L-DOPA in Retinal Disease and Potential Mechanisms

The mechanism of the sustained benefit of L-DOPA on rod-driven inner retinal function is currently unknown. L-DOPA is a DA precursor that is converted to DA in both the central and peripheral nervous systems. L-DOPA has been utilized extensively, and it has been US Food and Drug Administration (FDA) approved for treating motor symptoms in Parkinson's disease since the 1970s.^57^^,^^58^ Carbidopa is a decarboxylase inhibitor commonly used to allow L-DOPA to cross the blood-brain barrier and is often used in combination with L-DOPA.^59^ The use of L-DOPA in retinal disease has been increasingly tested, although still understudied.

L-DOPA appears to have a beneficial effect on several retinal diseases. In retrospective studies, L-DOPA was associated with a lower risk of patients progressing from non-neovascular age-related macular degeneration (AMD) to neovascular AMD (nAMD), as well as lowering the risk of developing geographic atrophy (GA).^60^^,^^61^ In addition, early treatment with L-DOPA improved visual acuity in patients with non-arteritic anterior ischemic optic neuropathy (NAION).^62^ Finally, patients with Parkinson's disease taking L-DOPA have greater choroidal thickness than patients not taking L-DOPA, suggesting a protective effect on the choriocapillaris which is observed to thin with progression to GA.^61^

The neuroprotective effects are unlikely to be due to a direct action of L-DOPA as the half-life of L-DOPA is 90 minutes^63^^,^^64^ and our data suggest that L-DOPA provides a sustained benefit after treatment in patients with diabetes. It seems likely that DA metabolism is dysfunctional at this early stage of diabetes, with decreases in DA noted in the retina,^31^ brain,^65^^,^^66^ and kidneys.^67^^–^^69^ This deficit may then progress into the loss of dopaminergic amacrine cells in the late stages of the disease.^57^ L-DOPA increases DA levels throughout the body, which may increase levels of DA and binding to DA receptors in retinal cells. However, our work in diabetic rodent models indicate that gene expression for the retinal DA receptors do not change with L-DOPA treatment;^31^^,^^56^ unfortunately, antibodies for the DA receptor are not available to confirm protein levels. Thus, we do not have evidence that L-DOPA is directly altering neuronal health.

An alternative explanation is that L-DOPA benefits the retinal vascular by acting as an anti-angiogenic agent. Additionally, the studies of the beneficial effects of L-DOPA in AMD have suggested that DA is increased by a process by which pigmentation in the retinal pigment epithelium (RPE) and choroid may activate melanin synthesis, increasing L-DOPA production and G-protein coupled receptor 143 (GPR143) signalling.^70^^,^^71^ GPR143 then upregulates pigment epithelium-derived factor (PEDF) and downregulates VEGF, two important factors in vascular growth and maintenance.^72^^,^^73^ In addition to RPE-mediated mechanisms, L-DOPA’s metabolic product, DA, may exert anti-angiogenic effects via DA receptors in the retina. DA acting upon D2 receptors can inhibit angiogenic signaling by promoting endocytosis of VEGF receptor-2, preventing vessel growth.^74^^–^^77^ Preventing D2 receptor activation resulted in increased vascular permeability, angiogenesis, and tumor growth.^78^ Vasculature structural changes are the hallmark of the clinical stages of DR and several studies have indicated that vascular function is abnormal in early stages of DR, prior to overt vascular pathology.^79^ Thus, in our studies, L-DOPA treatment may be neuroprotective to dysfunctional vasculature which improves neuronal function. Our current studies are examining potential correlations between rod-driven OP delays and early vascular dysfunction in diabetic rodents and also investigating altered microvascular changes with OCTA in clinical trials (NCT05132660).

### Retinal Structural Changes With Early-Stage DR

The relationship between retinal structure and function in DR is not entirely understood. In our study, we saw few retinal structure differences between the control and the DM group. This finding is consistent with a previous study that found no structural differences between non-diabetic participants and participants with diabetes when correcting for age, race, and gender in a multiethnic population.^80^ One possibility is that retinal structure was normal in the DM group, as this group had normal OP ITs and thus normal neuronal function.

Although we have examined the effects of L-DOPA on diabetic retinal function, the effects on retinal structure in the diabetic retina are unknown.^20^ In our study, we observed significant thinning of the OPL across the entire retina in the DM + L-DOPA group compared to the DM group, driven mainly by the outer region. The literature on OPL thickness changes with diabetes are mixed with reports of thinning,^81^ thickening,^82^ and no difference.^33^ In this analysis, the DM + L-DOPA group received L-DOPA treatment due to a delay in ERG ITs. Thus, any structural differences could be due to delayed ERG ITs and/or the effect of L-DOPA.

In contrast, we observed significant thickening in the outer region of the GCL in the DM compared to the DM + L-DOPA group. This result could be an early sign of DME in patients without signs of DR, as seen in other studies.^83^^,^^84^ Thus, it may suggest that L-DOPA helps to prevent DME because the group that did not receive L-DOPA had a thicker outer GCL, and the group that received L-DOPA had GCL thickness within the 95% CI of the healthy controls. With our small group size, we were not able to confirm this hypothesis in this study. The difference between GCL outer region thickness in DM and DM + L-DOPA could also be due to variability in blood pressure, which we did not analyze. One study found that increased ganglion cell complex thickness was associated with uncontrolled hypertension.^85^ A few studies have reported a decrease in the GCL and GCL/IPL complex.^36^^,^^86^^,^^87^ A limitation to the current study is that there were no OCTs taken at baseline, thus it is plausible that because the DM + L-DOPA group had significantly delayed OPs, that structural changes were present prior to the L-DOPA treatment.

### Retinal Structure and Function Relationships Were Altered With L-DOPA Treatment

The relationship between retinal structure and function in DR has been investigated with OCT and functional testing, such as visual field tests, visual acuity, and contrast sensitivity.^88^^–^^90^ One previous clinical study investigated the relationships between full-field ERGs using bright flash stimuli and 30-Hz flicker with changes in retinal thickness.^91^ To the best of our knowledge, this is the first clinical study to show correlations between full-field ERGs with dim-flash stimuli and retinal thickness changes. Although reports of direct associations between specific retinal layer thickness measurements and their ERG functional effects in diabetes are limited in the literature, it is well known that retinal function changes are associated with retinal structure changes, like loss of ERG a-wave and reduction in photoreceptor layer thickness. Thus, we present these novel findings of a potential relationship between retinal function and structure as a possible opportunity to provide valuable clinical information for predicting DR progression or informing disease management strategies. Surprisingly, the control and DM + L-DOPA groups were very similar among all regions, suggesting that L-DOPA treatment may benefit the patients with diabetes by maintaining a more normal structure-function relationship. Interestingly, the layers of the DM group that were significantly correlated with IT, ONL, and INL, contribute to the origin of the ERG response. The ONL contains photoreceptors that synapse in the OPL, which generate the a-wave.^92^ The INL contains ON bipolar and amacrine cells, which generate the b-wave and OPs, respectively.^93^^,^^94^

The positive correlation observed between the DM group's ONL center and OP 2 IT could be due to early onset DME or moderate NPDR.^95^ Consistent with other studies, significant thickening was found in the ONL center, which is typically where DME starts.^82^^,^^96^^–^^98^ One study reported ONL thickness increased in people with diabetes and no retinopathy compared with healthy controls.^81^ Another study found a significant increase in ONL thickness in subjects with moderate DR, absent of DME, compared to mild DR.^33^ These studies support our result that a thicker ONL center could be associated with a slower OP 2 IT.

The mechanisms behind the correlations between the DM groups inner and outer regions with OP 1 IT are unknown. However, some findings may suggest that the negative correlation between the DM groups OPL inner region and OP 1 IT could potentially be a result of hypoxia.^99^^–^^105^ Additionally, previous literature suggests that the positive correlation found between the DM groups INL outer region and OP 1 IT may be attributed to early development of DME, microaneurysms, or swelling of Muller cells.^82^^,^^95^^,^^106^^–^^109^

We hypothesize that these correlations in the DM group could be associated with adverse side effects and ocular complications of diabetes, such as DR, hypoxia, or DME. The control and DM + L-DOPA treatment groups do not show these strong correlations. This supports the idea that L-DOPA has a protective effect on the retina because the untreated DM group was the only group to have strong correlations between structure and function.

### Do Dim-Flash OP Delays Predict Clinically Recognized DR Fundus Pathology?

These results do not provide sufficient data to determine if dim-flash OP delays are predictive for retinal vascular pathology. In the DM group, 50% of the participants developed mild NPDR, whereas 43% of the participants in the DM + L-DOPA group had mild NPDR, moderate NPDR, or DME. The number of participants in these groups was small (*n* = 6 and *n* = 14, respectively), making it difficult to make any definitive conclusions. However, it is interesting to note that more individuals in the DM + L-DOPA group, which had significant dim-flash OP delays at baseline, had more advanced stages of DR, potentially suggesting that this inner retinal dysfunction may predict long-term structural changes. To further investigate the effects of L-DOPA on the development of clinical signs of DR, future studies should be conducted following individuals for a longer period of time.

### Limitations

All participants were recruited from the Joseph Maxwell Cleland Atlanta VA Medical Center, which restricted the ability to achieve race and sex balance within the study population. Additionally, given the age demographic of the participants and the extended 5-year follow-up period, participant loss over time was a significant factor, as many participants either passed away or relocated, resulting in a reduced sample size. Furthermore, because participants were drawn from the Motz et al. (2020) study, their initial group assignments had to be accounted for, leading to an unmasked study design. Future research should prioritize blinded, randomized controlled trials with larger, more demographically diverse cohorts to mitigate these limitations and assess potential race- or sex-based differences in treatment response.

Whereas the findings suggest that dim-flash OP delays returned to baseline within 4.5 to 6 years in the treated group, further research is needed to determine the duration of L-DOPA’s protective effects in individuals with diabetes. Longitudinal studies with more frequent follow-ups should be conducted to elucidate the persistence of these benefits. Additionally, the potential synergistic effects of DA agonists, such as bromocriptine, previously shown to reduce retinal vessel permeability, should be explored in combination with L-DOPA treatment.^110^ Incorporating optical coherence tomography angiography (OCT-A) in future studies would also allow for a more detailed assessment of deeper retinal vascular changes associated with DR.

## Conclusions

Over a 5-year period, OP IT increased but did not surpass baseline delays. This finding suggests two potential explanations. The effects from short-term treatment of L-DOPA may have long-lasting effects. Alternatively, OP IT delays in response to dim-flash may develop at a reduced rate in humans with DM than expected through observations seen in diabetic rodent models. To elucidate which conclusion is correct, randomized controlled, clinical studies with patients with DM receiving L-DOPA need to be conducted. Furthermore, we observed moderate strength correlations between structure and function in the DM group, whereas L-DOPA treatment appeared to benefit the DM + L-DOPA group with weak correlations that were similar to the control group. These findings highlight the potential of OP biomarkers in personalized disease management and predicting progression of disease, offering insights into an individual's unique disease trajectory and response to treatment.

## Supplementary Material

Supplement 1
