# Supplementary material for: Sustained Benefit of Short-Term Levodopa Treatment on Inner Retinal Function in Patients With Diabetes
Source: Transl Vis Sci Technol. 2025 Sep 2;14(9):5. doi: 10.1167/tvst.14.9.5 (PMC12410260; doi:10.1167/tvst.14.9.5)
Supplement: Supplement 1 [file tvst-14-9-5_s001.pdf]

Sustained benefit of short-term levodopa treatment on inner retinal function in patients with diabetes, Moore et al., Transl Vis Sci Technol 2025

Supplementary Material

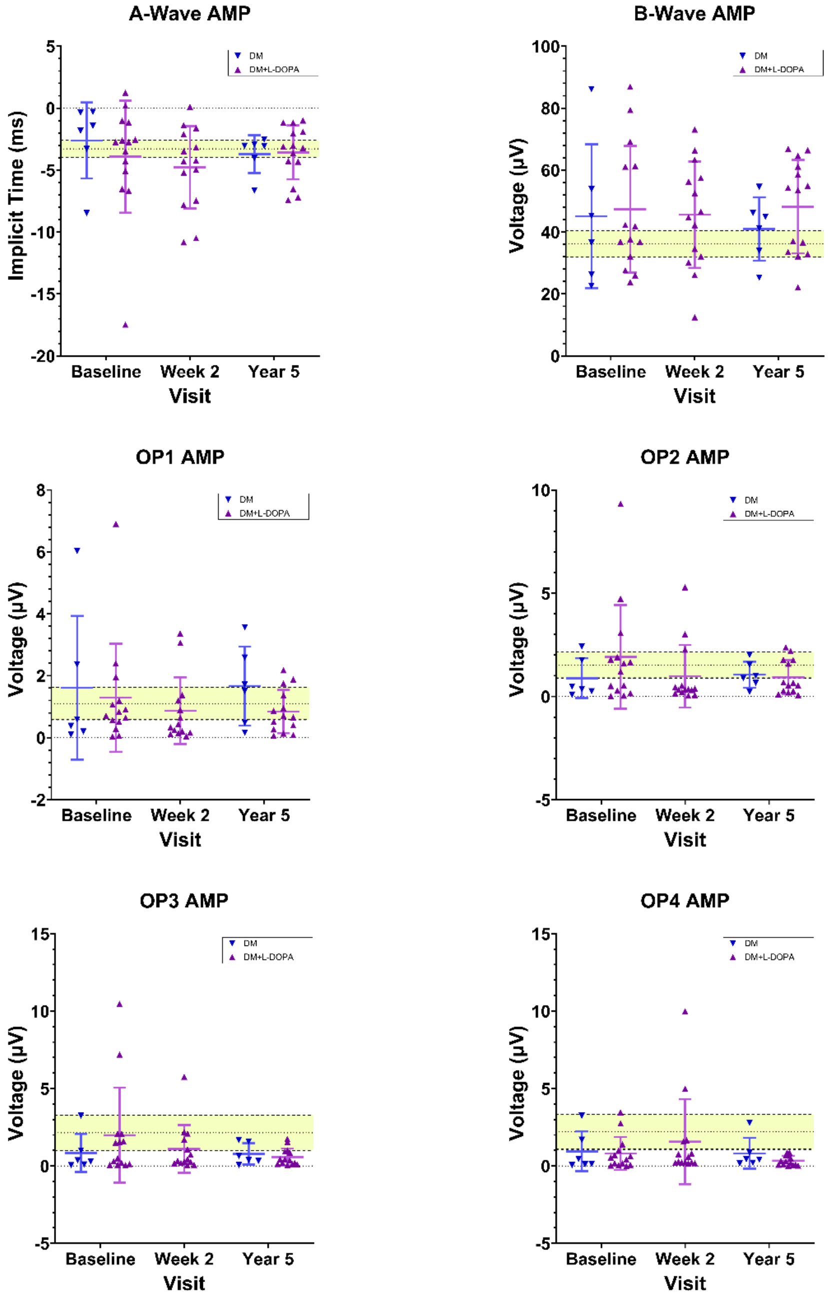

Figure S1 — Mean (±SD) ERG OP amplitudes in DM and DM+L-DOPA groups at baseline, Week 2, and Year 5 exposed to dim flash stimuli. **A)** a-wave amplitude, **B)** b-wave amplitude, **C)** OP1 amplitude, **D)** OP2 amplitude, **E)** OP3 amplitude, and **F)** OP4 amplitude. The yellow horizontal band indicates the normative 95% confidence interval derived from healthy controls. A Wilcoxon rank-sum test was used to compare the DM and DM+L-DOPA groups at baseline and Year 5. No significant differences were found between groups ($p < 0.05$ *, *$p < 0.01$ **, $p < 0.001$ ***). A one-way repeated measures ANOVA was performed within the DM+L-DOPA group across baseline, Week-2, and Year-5, showing no significant differences across visits.

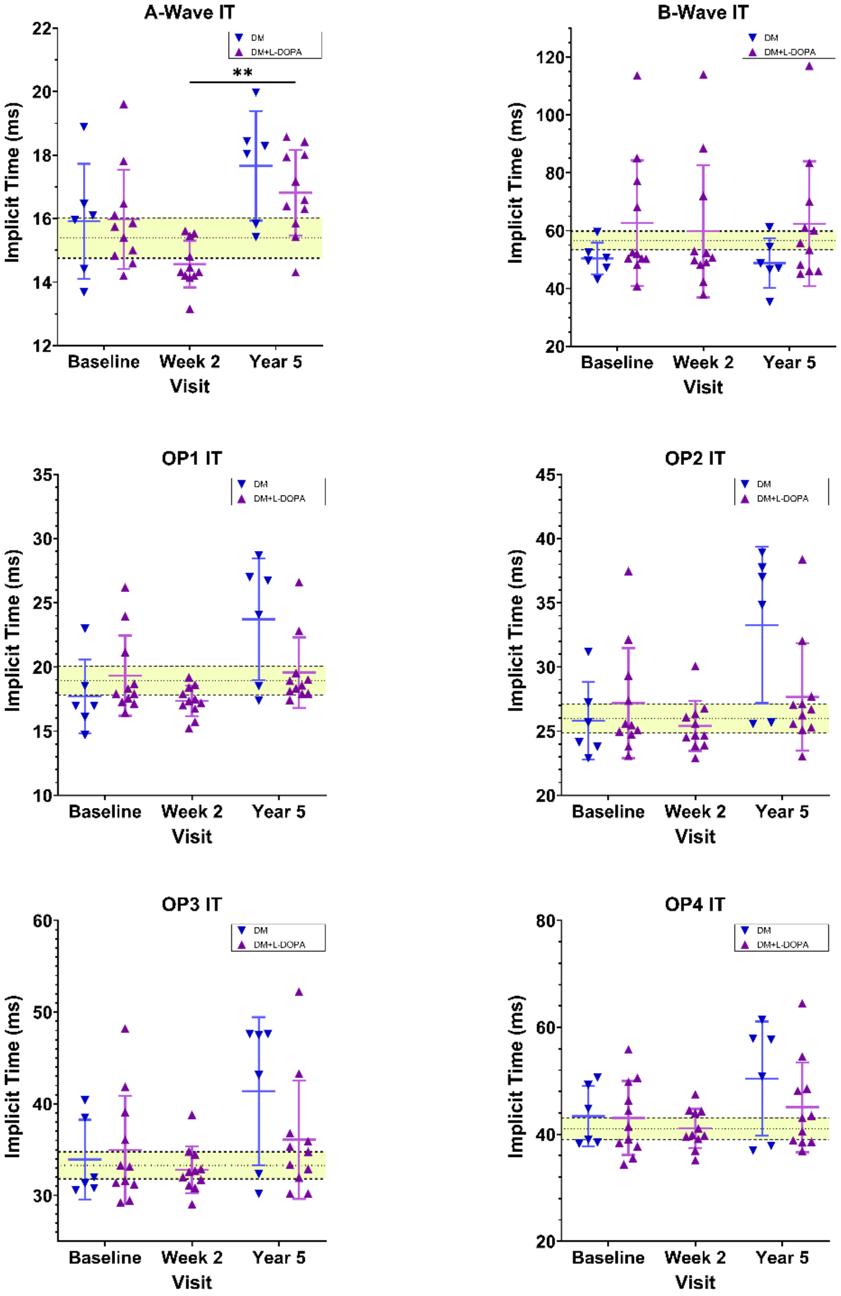

Figure S2 — Mean (±SD) ERG OP IT in DM and DM+L-DOPA groups at baseline, Week 2, and Year 5 exposed to bright flash stimuli. **A)** a-wave IT, **B)** b-wave IT, **C)** OP1 IT, **D)** OP2 IT, **E)** OP3 IT, and **F)** OP4 IT. The yellow horizontal band indicates the normative 95% confidence interval derived from healthy controls. A Wilcoxon rank-sum test was used to compare the DM and DM+L-DOPA groups at baseline and Year 5. No significant differences were found between groups ($p < 0.05$ *, *p < 0.01 **, $p < 0.001$ ***). A one-way repeated measures ANOVA was performed within the DM+L-DOPA group across baseline, Week-2, and Year-5. A-wave IT showed a significant regression in timing from Week-2 to Year-5, becoming more delayed after the discontinuation of L-DOPA ($p = 0.0017$).

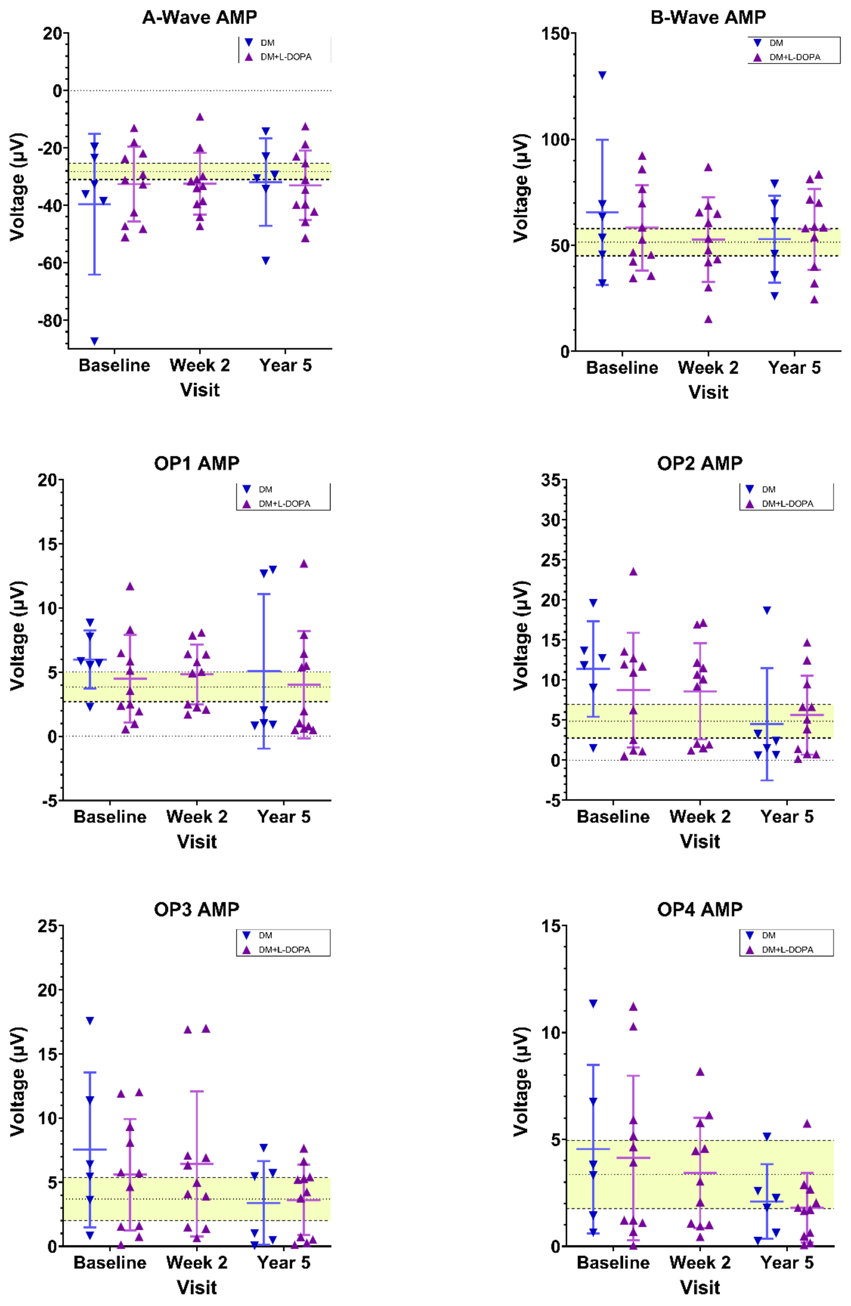

Figure S3 — Mean (±SD) ERG OP amplitudes in DM and DM+L-DOPA groups at baseline, Week 2, and Year 5 exposed to bright flash stimuli. **A)** a-wave amplitude, **B)** b-wave amplitude, **C)** OP1 amplitude, **D)** OP2 amplitude, **E)** OP3 amplitude, and **F)** OP4 amplitude. The yellow horizontal band indicates the normative 95% confidence interval derived from healthy controls. A Wilcoxon rank-sum test was used to compare the DM and DM+L-DOPA groups at baseline and Year 5. No significant differences were found between groups ($p < 0.05$ *, *$p < 0.01$ **, $p < 0.001$ ***). A one-way repeated measures ANOVA was performed within the DM+L-DOPA group across baseline, Week-2, and Year-5, showing no significant differences across visits.

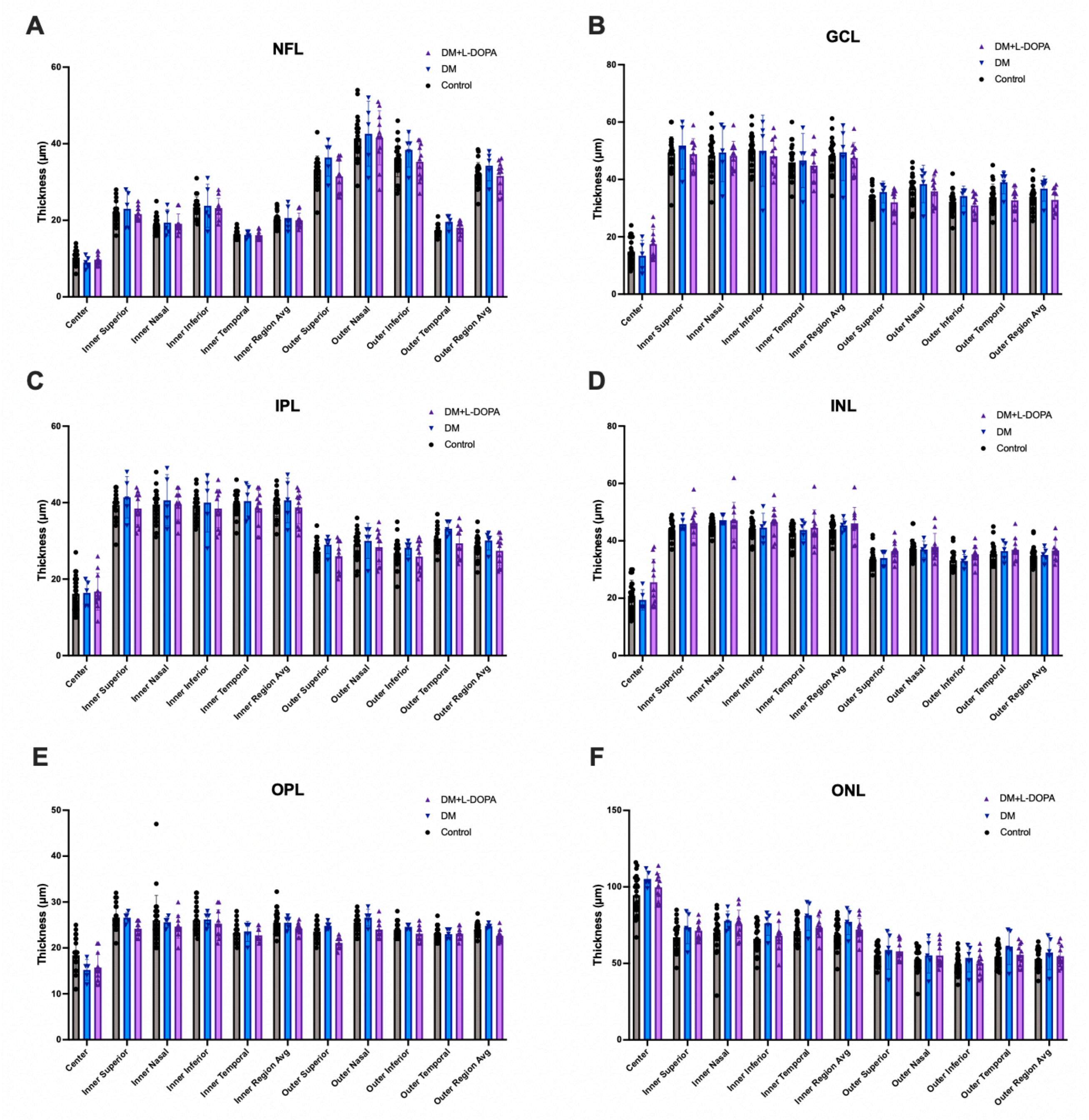

Figure S4 — Thickness (mean ± SD) of Macular ETDRS regions compared between Control (n = 25), DM (n = 5), and DM+L-DOPA (n = 11) for retinal layers **A)** NFL **B)** GCL **C)** IPL **D)** INL **E)** OPL **F)** ONL. There was no significance comparing the means across groups (Kruskal-Wallis test: $p < 0.05$ *, $p < 0.01$ **, $p < 0.001$ ***).

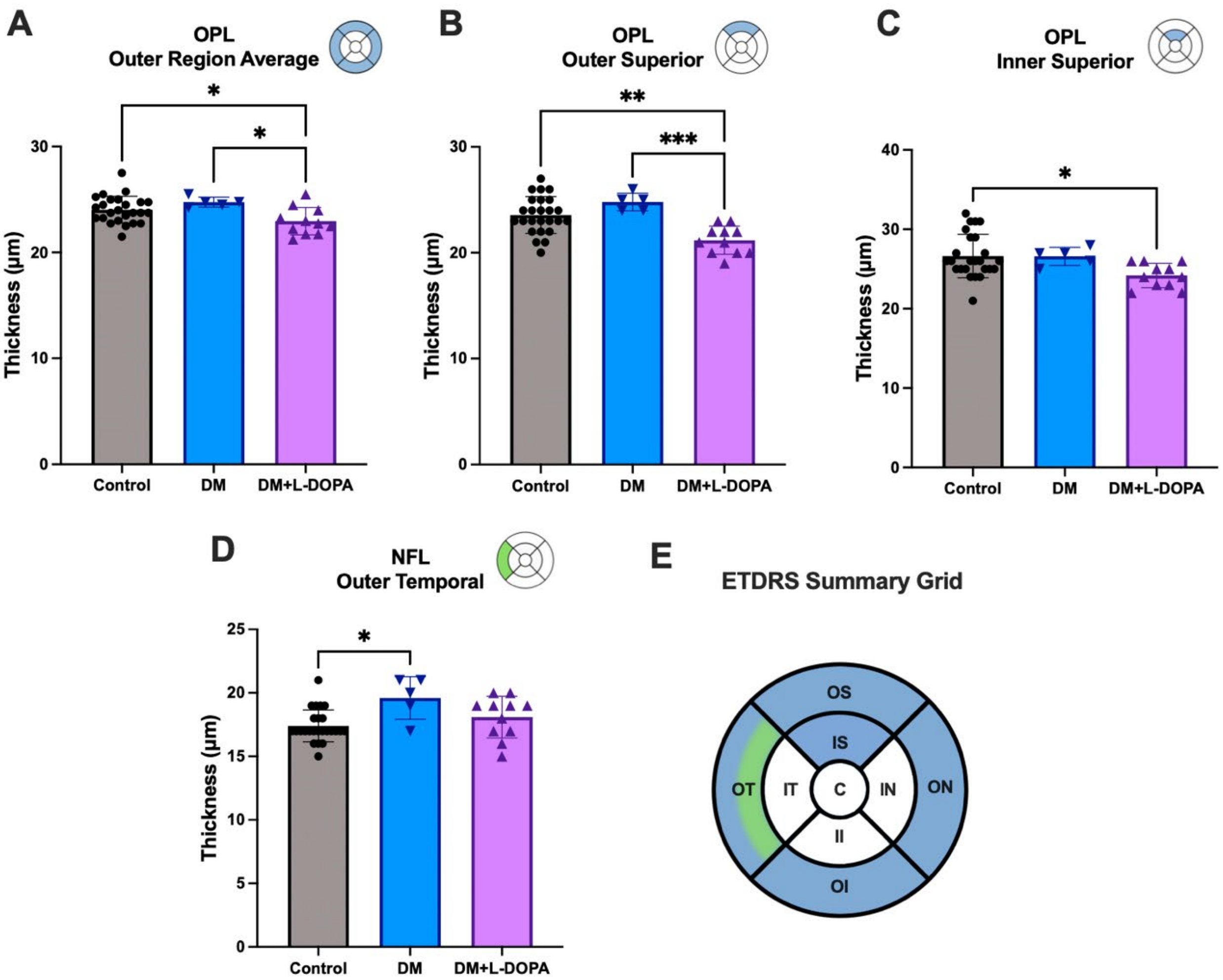

Figure S5 — OCT retinal thickness at Year-5 in diabetic participants (DM, n = 5), diabetic participants who received levodopa treatment (DM+L-DOPA, n = 11), and a control group (n = 25). **A)** OPL outer region average **B)** OPL outer superior **C)** OPL inner superior **D)** NFL outer temporal superior **(**Kruskal-Wallis test: $p < 0.05$ *, $p < 0.01$ **, $p < 0.001$ ***). **H)** ETDRS Summary Grid of retinal thickness changes. Green represents summary of NFL retinal thickness changes and blue represents summary OPL retinal thickness changes.

When compared to the control group, the OPL outer region average of the DM+L-DOPA group was significantly thinner (Ctrl: 24.04 ± 1.27 μm, DM: 24.75 ± 0.47 μm, and DM+L-DOPA: 22.95 ± 1.29 μm, $p < 0.05$). Additionally, the outer superior region of the OPL was significantly thinner in the DM+L-DOPA compared to the Ctrl (Ctrl: 23.56 ± 1.73 μm, DM: 24.80 ± 0.84 μm, and DM+L-DOPA: 21.18 ± 1. 3 μm, $p < 0.01$). The DM+L-DOPA group also had a significantly thinner OPL inner superior region than the Ctrl (Ctrl: 26.64 ± 2.74 μm, DM: 26.60 ± 1.14 μm, and DM+L-DOPA: 24.18 ± 1.54 μm, $p < 0.05$). Additionally, both diabetic groups had a larger NFL outer temporal region, however, only the DM group reached significance (Ctrl: 17.40 ± 1.26 μm, DM: 19.60 ± 1.67 μm, and DM+L-DOPA: 18.09 ± 1.642 μm, $p < 0.05$).

Retinal Layer	Macular ETDRS Region	Control (*n* = 25)	DM (*n* = 5)	DM+L-DOPA (*n* = 11)	*P*-value
NFL					
	Inner Region	20.2 ± 1.8	20.6 ± 3.4	20.0 ± 1.9	0.8426
	Outer Region	32.0 ± 3.2	34.3 ± 4.0	31.6 ± 3.9	0.3653
	Total Layer	24.3 ± 2.0	25.4 ± 3.2	24.0 ± 2.5	0.5317
GCL					
	Inner Region	48.3 ± 5.46	49.5 ± 9.9	47.5 ± 5.4	0.6213
	Outer Region	34.0 ± 4.0	36.8 ± 4.4	32.9 ± 4.3	0.1599
	Total Layer	38.2 ± 4.1	39.8 ± 6.7	37.6 ± 4.4	0.4046
INL					
	Inner Region	44.0 ± 2.7	45.4 ± 2.2	46.1 ± 5.6	0.5211
	Outer Region	34.8 ± 3.1	35.1 ± 2.6	36.6 ± 3.6	0.2518
	Total Layer	37.4 ± 2.4	37.9 ± 1.8	39.6 ± 4.4	0.2364
IPL					
	Inner Region	39.5 ± 3.2	40.6 ± 5.8	38.8 ± 4.3	0.8993
	Outer Region	28.4 ± 2.9	30.1 ± 2.5	27.4 ± 3.9	0.2806
	Total Layer	32.0 ± 2.6	33.2 ± 3.9	31.3 ± 3.7	0.5602
OPL					
	Inner Region	25.5 ± 2.5	25.5 ± 1.4	24.2 ± 1.2	0.6163
	Outer Region	24.0 ± 1.3	24.8 ± 0.5	22.8 ± 1.2	**0.0150***
	Total Layer	24.0 ± 1.8	24.0 ± 0.9	22.6 ± 1.1	0.0557
ONL					
	Inner Region	68.6 ± 8.8	77.2 ± 8.4	72.1 ± 7.3	0.1310
	Outer Region	53.0 ± 6.5	57.2 ± 11.2	54.7 ± 7.1	0.5004
	Total Layer	64.6 ± 7.8	71.4 ± 8.4	67.4 ± 6.8	0.2323

Data presented as (mean ± SD)
*Kruskal-Wallis Test

Table S1 — Retinal thickness (mean ± SD) of Macular ETDRS inner and outer region averages and total layer compared between all layers of Control (n = 25), DM (n = 5), and DM+L-DOPA (n = 11). The outer region of the OPL was significantly different between groups ($p < 0$ .05; Kruskal-Wallis test: $p < 0.05$ *, $p < 0.01$ **, $p < 0.001$).

Retinal Layer	Macular ETDRS Region	Control (*n* = 25)	DM (*n* = 5)	DM+L-DOPA (*n* = 11)	*P*-value
NFL					
	Center	10.3 ± 1.8	9.0 ± 1.6	9.7 ± 1.4	0.2153
	Inner superior	22.0 ± 2.7	23 ± 4.8	21.6 ± 1.9	0.7484
	Inner nasal	18.9 ± 2.0	19.4 ± 3.4	19.1 ± 2.6	0.9841
	Inner inferior	23.4 ± 2.7	23.8 ± 5.6	23.2 ± 2.6	0.9529
	Inner temporal	16.4 ± 1.1	16.2 ± 0.8	16.1 ± 1.1	0.7011
	Outer superior	33.0 ± 3.7	36.4 ± 4.9	31.5 ± 4.5	0.1641
	Outer nasal	41.4 ± 5.4	42.6 ± 8.5	41.5 ± 7.1	0.8332
	Outer inferior	36.0 ± 4.6	38.4 ± 4.5	35.3 ± 4.8	0.3908
	Outer temporal	17.4 ± 1.3	19.6 ±1.7	18.1 ± 1.6	**0.0228***
GCL					
	Center	14.8 ± 4.5	13.4 ± 5.4	17.5 ± 5.1	0.3415
	Inner superior	49.1 ± 5.8	51.8 ± 8.2	48.8 ± 5.4	0.5856
	Inner nasal	48.3 ± 6.4	49.4 ± 10.2	48.2 ± 5.1	0.8179
	Inner inferior	48.9 ± 5.5	50.0 ± 12.4	48.1 ± 6.1	0.4674
	Inner temporal	46.0 ± 5.8	46.6 ± 9.4	44.8 ± 5.7	0.7711
	Outer superior	33.0 ± 3.7	35.6 ± 3.8	32.0 ± 4.2	0.2057
	Outer nasal	36.8 ± 4.6	38.4 ± 6.5	35.8 ± 5.1	0.4347
	Outer inferior	32.2 ± 3.8	34.2 ± 3.5	30.9 ± 4.0	0.1714
	Outer temporal	33.8 ± 4.6	39.0 ± 4.1	32.7 ± 4.1	0.0555
INL					
	Center	20.8 ± 5.4	19.4 ± 3.6	25.5 ± 8.0	0.2277
	Inner superior	43.7 ± 3.2	45.8 ± 2.2	46.2 ± 5.3	0.1728
	Inner nasal	45.4 ± 2.6	47.2 ± 1.6	47.1 ± 6.3	0.3955
	Inner inferior	44.2 ± 2.9	44.6 ± 5.0	46.5 ± 5.2	0.2467
	Inner temporal	42.7 ± 3.3	43.8 ± 3.3	44.5 ± 6.2	0.7320
	Outer superior	33.6 ± 3.3	34.0 ± 2.7	36.4 ± 3.4	0.0854
	Outer nasal	37.2 ± 3.4	37.0 ± 2.9	37.9 ± 4.7	0.9995
	Outer inferior	33.2 ± 3.1	33.0 ± 2.2	35.3 ± 3.2	0.1158
	Outer temporal	35.4 ± 3.4	36.4 ± 3.4	36.9 ± 4.0	0.3265
IPL					
	Center	16.2 ± 4.4	16.4 ± 3.3	16.7 ± 4.8	0.9064
	Inner superior	39.4 ± 3.5	41.4 ± 5.4	38.5 ± 4.2	0.5409
	Inner nasal	39.5 ± 3.9	40.6 ± 6.7	39.5 ± 4.0	0.9527
	Inner inferior	39.3 ± 3.4	40.0 ± 7.7	38.5 ± 5.0	0.6739
	Inner temporal	39.8 ± 2.9	40.4 ± 4.6	38.5 ± 4.7	0.7323
	Outer superior	27.2 ± 2.9	29.0 ± 2.3	26.0 ± 4.2	0.3264
	Outer nasal	29.0 ± 3.5	30.0 ± 4.6	28.4 ± 4.5	0.5271
	Outer inferior	26.8 ± 3.5	28.2 ± 1.9	25.9 ± 4.0	0.4963
	Outer temporal	30.6 ± 2.6	33.2 ± 1.8	29.4 ± 4.2	0.0751
OPL					
	Center	18.4 ± 3.7	15.2 ± 2.3	15.7 ± 3.6	**0.0406***
	Inner superior	26.6 ± 2.7	26.6 ± 1.1	24.2 ± 1.5	**0.0119***
	Inner nasal	26.0 ± 5.4	25.6 ± 1.1	24.6 ± 2.5	0.6713
	Inner inferior	26.0 ± 3.0	26.2 ± 1.6	25.3 ± 3.0	0.6750
	Inner temporal	23.3 ± 1.9	23.6 ± 2.2	22.7 ± 1.5	0.5240
	Outer superior	23.6 ± 1.7	24.8 ± 0.8	21.2 ± 1.3	**0.0002***
	Outer nasal	25.5 ± 1.9	26.6 ± 1.8	24.1 ± 1.8	**0.0249***
	Outer inferior	24.0 ± 1.6	24.6 ± 0.5	23.0 ± 1.6	0.1439
	Outer temporal	23.1 ± 1.4	23.0 ± 1.0	22.9 ± 1.4	0.9949
ONL					
	Center	94.6 ± 12.6	105.2 ± 5.2	99.7 ± 8.9	0.1338
	Inner superior	67.1 ± 9.1	73.6 ± 10.7	71.4 ± 6.1	0.1958
	Inner nasal	70.3 ± 11.6	77.8 ± 6.4	75.5 ± 9.3	0.1778
	Inner inferior	66.1 ± 8.9	76.2 ± 7.9	68.0 ± 8.9	0.0789
	Inner temporal	71.1 ± 8.0	81.2 ± 9.6	73.4 ± 6.5	0.0914
	Outer superior	55.2 ± 6.2	58.8 ± 12.6	57.8 ± 6.8	0.4066
	Outer nasal	52.6 ± 7.4	55.2 ± 11.5	55.2 ± 7.9	0.6307
	Outer inferior	49.7 ± 6.5	53.6 ± 9.2	50.1 ± 7.5	0.4981
	Outer temporal	54.4 ± 7.0	61.0 ± 11.7	55.6 ± 7.1	0.4124

Data presented as (mean ± SD)
*Kruskal-Wallis Test

Table S2 — Retinal thickness (mean ± SD) of Macular ETDRS regions compared between all layers of Control (n = 25), DM (n = 5), and DM+L-DOPA (n = 11). There was a significant difference across groups in the NFL outer temporal ($p < 0.05$), the OPL center ($p < 0.05$), inner superior ($p < 0.05$), outer superior ($p < 0.001$), and outer nasal ($p < 0.05$; Kruskal-Wallis test: $p < 0.05$ *, $p < 0.01$ **, $p < 0.001$).
